# Virtuelle Welten in der Fernlehre – Eine Studie der Fernhochschule AKAD University

**DOI:** 10.1365/s40702-023-00976-y

**Published:** 2023-04-26

**Authors:** Heidi Rinn, Daniel Markgraf, Susanne Robra-Bissantz

**Affiliations:** 1 Institute for Digital Expertise and Assessment (IDEA), AKAD University, Stuttgart, Deutschland; 2 Institut für Wirtschaftsinformatik, TU Braunschweig, Braunschweig, Deutschland

**Keywords:** Virtuelle Welt, 3D Lernumgebung, Avatar, E‑Learning, Hochschule, Fernlehre, Virtual world, 3D learning environment, Avatar, E‑Learning, University, Distance learning

## Abstract

Die Corona-Pandemie hat die Welt der Hochschullehre in vielerlei Hinsicht verändert. In Folge der Beschränkungen häufig eingesetzte Videokonferenzlösungen erwiesen sich für einige Themen als guter Ersatz bei gleichzeitiger Erhöhung der örtlichen Flexibilität. Es wurde aber auch offensichtlich, dass es Lehrformate gibt, die per Videokonferenz nicht ideal umsetzbar sind. Dazu zählen u. a. Seminare mit starkem Fokus auf Gruppenarbeit oder Kursinhalte, die viel Hilfestellung durch den Dozierenden erfordern. Virtuelle Welten werden in diesen Fällen aufgrund ihrer sozialen und räumlichen Präsenz als potenziell bessere Alternative zu Videokonferenzen identifiziert. Als besonders problematisch werden an der Institution, einer deutschen Fernhochschule, zwei Seminare identifiziert, nämlich ein Programmierseminar und ein Wirtschaftsplanspiel. Hierfür wird eine prototypische Lösung mit der virtuellen Welt von TriCAT spaces explorativ gestaltet, durchgeführt und evaluiert. Wir stellen vor, wie diese beiden Seminare erfolgreich in eine virtuelle Welt portiert werden können. Die Evaluierung der Stichprobe (*N* = 23) zeigt, dass die Umsetzung in 3D demselben Seminar in Zoom in Bezug auf Flow-Erleben zwar nur leicht überlegen ist, allerdings trotzdem die meisten die 3D-Variante vorziehen. Bei der Beobachtung der 3D-Seminare identifizieren wir Verbesserungsmöglichkeiten für nachfolgende Prototypen und legen den Grundstein für vertiefende Forschungen.

## Abstract (en)

The Corona pandemic changed the world of higher education teaching in many ways. Video conferencing solutions commonly used in the wake of the restrictions proved to be a good substitute for some topics while increasing local flexibility. However, it also became apparent that there are teaching formats that are not ideally implementable via videoconferencing. These include seminars with a strong focus on group work or course content that requires a lot of assistance from the instructor. Virtual worlds are identified as a potentially better alternative to video conferencing in these cases due to their social and spatial presence. Two seminars are identified as particularly problematic at the institution, a German distance learning university, namely a programming seminar and a business simulation. For this purpose, a prototypical solution using the virtual world of TriCAT spaces is exploratively designed, implemented, and evaluated. We present how these two seminars can be successfully ported to a virtual world. The evaluation of the sample (*N* = 23) shows that although the implementation in 3D is only slightly superior to the same seminar in Zoom in terms of flow experience, most prefer the 3D variant. In observing the 3D seminars, we identify opportunities for improvement for subsequent prototypes and lay the groundwork for more in-depth research.

## Einleitung und Problemstellung

Die Einschränkungen der Corona-Pandemie haben Hochschulen kurzfristig zu einer Umstellung von Präsenzunterricht hin zu Online-Unterricht gezwungen. Eine vollständige Rückkehr zu Präsenzunterricht zeichnet sich aber auch nach der Pandemie nicht ab (Luebcke et al. 2022). Es stellt sich daher die Frage nach effizienter Gestaltung von Online-Lehrformaten.

Die AKAD University ist eine private Fernhochschule, deren Studierende größtenteils Vollzeit berufstätig und örtlich verstreut sind. Das Studienmodell wurde bereits vor COVID-19 kontinuierlich digitalisiert, Präsenzseminare sind eher die Ausnahme und wurden gezielt dort eingesetzt, wo Videokonferenzlösungen als schlechte Alternative eingestuft wurden: So z. B. bei Gruppenarbeiten, die eine enge Zusammenarbeit der Studierenden voraussetzen oder bei Programmierseminaren, bei denen Unterstützung durch „über die Schulter schauen“ als wertvoll angesehen wurde. Dozierende beklagen zudem die Inaktivität von Studierenden in Online-Seminaren (Blumentritt et al. 2020).

Virtuelle Welten versprechen „immersives Lernen“ mit räumlicher und sozialer Präsenz. Laut einer Studie des MMB-Instituts besteht im Unternehmenskontext sogar ein leichter Trend hin zu „Lernumgebungen in virtuellen 3D-Welten“ (mmb Institut GmbH 2022). Professionelle Plattformen wie *TriCAT spaces* oder *ENGAGE* haben frühe Lösungen wie *Second Life* oder *OpenSim* auch an den Hochschulen abgelöst (Lecon und Koot 2015; Lückemeyer 2015) und die Erfahrungen damit sind positiv. Diese empirischen Studien belegen die Eignung für u. a. Lehrveranstaltungen zur Programmierung oder Grundlagen der Mathematik, sowie Gruppenarbeiten und geben Gestaltungsempfehlungen.

Die Forschungsfrage, die sich aus den bisherigen Forschungen ableitet, lautet, wie sich vorhandene Gestaltungsaspekte um weitere Anwendungsszenarien in der Fernlehre und um nicht-technische Zielgruppen erweitern lassen.

Ziel ist die Gestaltung von Lehrveranstaltungen in einer virtuellen Welt, sowie deren Evaluation gegen die bisherigen Online-Lehrveranstaltungen in Zoom, einerseits für eine technische, andererseits für eine nicht-technische Zielgruppe. Dabei gilt es nicht nur Unterschiede in der Wahrnehmung der Zielgruppen herauszufinden, sondern idealerweise auch zu begründen. Dazu werden zunächst geeignete Seminare identifiziert, gestaltet, umgesetzt und evaluiert. Die Evaluierung umfasst die Beobachtung und eine Online-Befragung. Die Ergebnisse werden diskutiert, kritisch hinterfragt und es werden Implikationen für Forschung und Praxis abgeleitet.

## Virtuelle Welten im Kontext der Hochschullehre

Virtuelle Welten können definiert werden als „Gemeinsame, simulierte Räume, die von ihren Bewohnern, die als Avatare dargestellt werden, mitgestaltet werden können. Diese Avatare vermitteln unsere Erfahrungen in diesem Raum, wenn wir uns bewegen, und mit Objekten oder anderen Avataren interagieren, mit denen wir ein gemeinsames Verständnis von der Welt in diesem Moment teilen“ (Girvan 2018, S. 1099).

Als wesentliche Eigenschaften einer virtuellen Welt gelten die soziale und räumliche Präsenz (Lecon und Koot 2015). Das Konzept der sozialen Präsenz geht zurück auf Short et al. (1976), die darunter die Wahrnehmung der anderen Personen und die damit verbundene Wahrnehmung der Beziehung zu den anderen Nutzern von digitalen Kommunikationsmedien verstehen. D. h. die Anforderungen an Kommunikationstechnologien gehen über die reine Informationsübertragung hinaus. Die räumliche Präsenz wird oft mit dem Gefühl beschrieben „sich tatsächlich dort“ zu fühlen und wird teilweise synonym zum Begriff „Immersion“ verwendet (Lecon und Herkersdorf 2014). Der Avatar sorgt im Vergleich zum Videobild für eine gewisse Anonymität, die sich in einer empirischen Studie positiv auf Kollaboration und Austausch, sowie Vernetzung, insbesondere bei eher introvertierteren Teilnehmern, ausgewirkt hat (Lecon und Koot 2015).

Das Flow-Erleben, das auf Csikszentmihalyi (1975) zurückgeht, wird in der Lehre als überaus bedeutsam erachtet und durch hohes Präsenzerleben gefördert (Zinn et al. 2016; Rodríguez-Ardura und Meseguer-Artola 2017). Es beschreibt den Zustand völligen Aufgehens in einer Tätigkeit. Flow hat einen positiven Einfluss auf die Motivation (Zinn et al. 2016), einem wesentlichen Erfolgsfaktor, insbesondere bei einer berufsbegleitenden Weiterbildung bzw. Studium.

Vor diesem Hintergrund lassen sich virtuelle Welten als vielversprechende Lösung für die Gestaltung von Online-Lehrveranstaltungen ausmachen, um neben der reinen inhaltlichen Vermittlung auch den sozialen Aspekten des Lernens gerecht zu werden. Aufgrund der positiven praktischen Erfahrungen aus dem Hochschulbereich (Lecon und Koot 2015; Lückemeyer 2015) und der mehrfachen Auszeichnung mit dem *eLearning Award*, wird die virtuelle Welt *TriCAT spaces* für die prototypische Umsetzung genutzt.

## Gestaltung der Lehrveranstaltungen

Zunächst wird der Ablauf der beiden ausgewählten Lehrveranstaltungen, nämlich Wirtschaftsplanspiel (WPS) und technisches Programmierseminar (TPS), allgemein beschrieben. Anschließend wird auf die Umsetzung in Zoom (2D) und TriCAT spaces (3D) eingegangen.

Das zweitägige TPS besteht aus den Elementen Frontalunterricht und Programmierübungen. Die Übungen erfolgen in Einzelarbeit und werden bei den Teilnehmern lokal ausgeführt, die entsprechende Entwicklungsumgebung muss vorab installiert werden. Zur Interaktion werden (per Umfragefunktion) Quizfragen gestellt. Diese werden als Multiple-Choice-Fragen in den Foliensatz des Dozierenden integriert, um sie methodenunabhängig (2D, 3D und Präsenz) nutzen zu können.

Das eintägige WPS erfolgt in Gruppenarbeit und ist Auftakt für vier Wochen Spielzeit und der anschließenden Anfertigung einer gemeinsamen Studienarbeit. Neben einer Einleitung (Frontalunterricht) und kurzen Zwischenbesprechungen in der Großgruppe, ist der Hauptanteil die Spielbearbeitung in Kleingruppen von 2–4 Teilnehmern. Die Kleingruppen führen im web-basierten Planspiel *TopSim* ein virtuelles Unternehmen und treten am simulierten Markt im Wettbewerb gegeneinander an.

Trotz der Wahlfreiheit der Modulreihenfolge im Fernstudium, stehen die Teilnehmer beider Veranstaltungen in aller Regel nicht am Anfang ihres Studiums, sondern haben das erste Drittel bereits absolviert. Zoom ist das Videokonferenztool, das hochschulweit zum Einsatz kommt und durch den hohen Online-Anteil den Studierenden früh im Studium begegnet. In 2D werden die Unterrichtspassagen über die Folienpräsentation und die Bildschirmfreigabe des Dozierenden realisiert, der in aller Regel seine Webcam aktiviert hat. Die Teilnehmenden haben die Webcam üblicherweise über weite Strecken deaktiviert. Bei lokalen Aufgaben kann immer nur einer der Teilnehmenden sein Problem für alle sichtbar teilen, um Unterstützung zu erhalten. Gruppenarbeiten nutzen die Gruppenfunktion des Videokonferenztools. Interaktionen werden über Umfragen oder Emojis initiiert. Der Chat wird beispielsweise genutzt, um Links zu Lehrmaterialien zu verteilen.

Die Konzeption zur Umsetzung in 3D folgte dem TPACK Modell, das für die Gestaltung von digitalen Lehrveranstaltungen neben den Wissensbereichen Technik, Pädagogik und Lehrinhalte insbesondere die Bedeutung der Schnittmengen dieser Wissensgebiete hervorhebt (Mishra und Koehler 2006). Darauf basierend wurde die Konzeption kollaborativ von drei Expert*innen entwickelt, wovon zwei die Fachbereiche Pädagogik und Technik und eine die Bereiche Pädagogik und Inhalte abdeckte. Die 2–3 Konzeptionsveranstaltungen fanden in der virtuellen Welt statt, um Einarbeitung der Dozierenden, Konzeption und Erprobung miteinander zu verbinden. So entstand auch die individualisierte Raumgestaltung über die Editorfunktion, die abgespeichert und am Tag der jeweiligen Veranstaltung wieder geladen werden konnte. Genutzt wurde das größte Raumszenario von TriCAT spaces (genannt *Premium*), das über drei Besprechungsräume unterschiedlicher Größe, eine Aula und einen Trainingsraum verfügt. Hinzu kommt ein sonnig gestalteter Außenbereich mit Sofainsel und Teich, wo im Hintergrund leises Vogelgezwitscher zu hören ist.

**Abb. 1:**
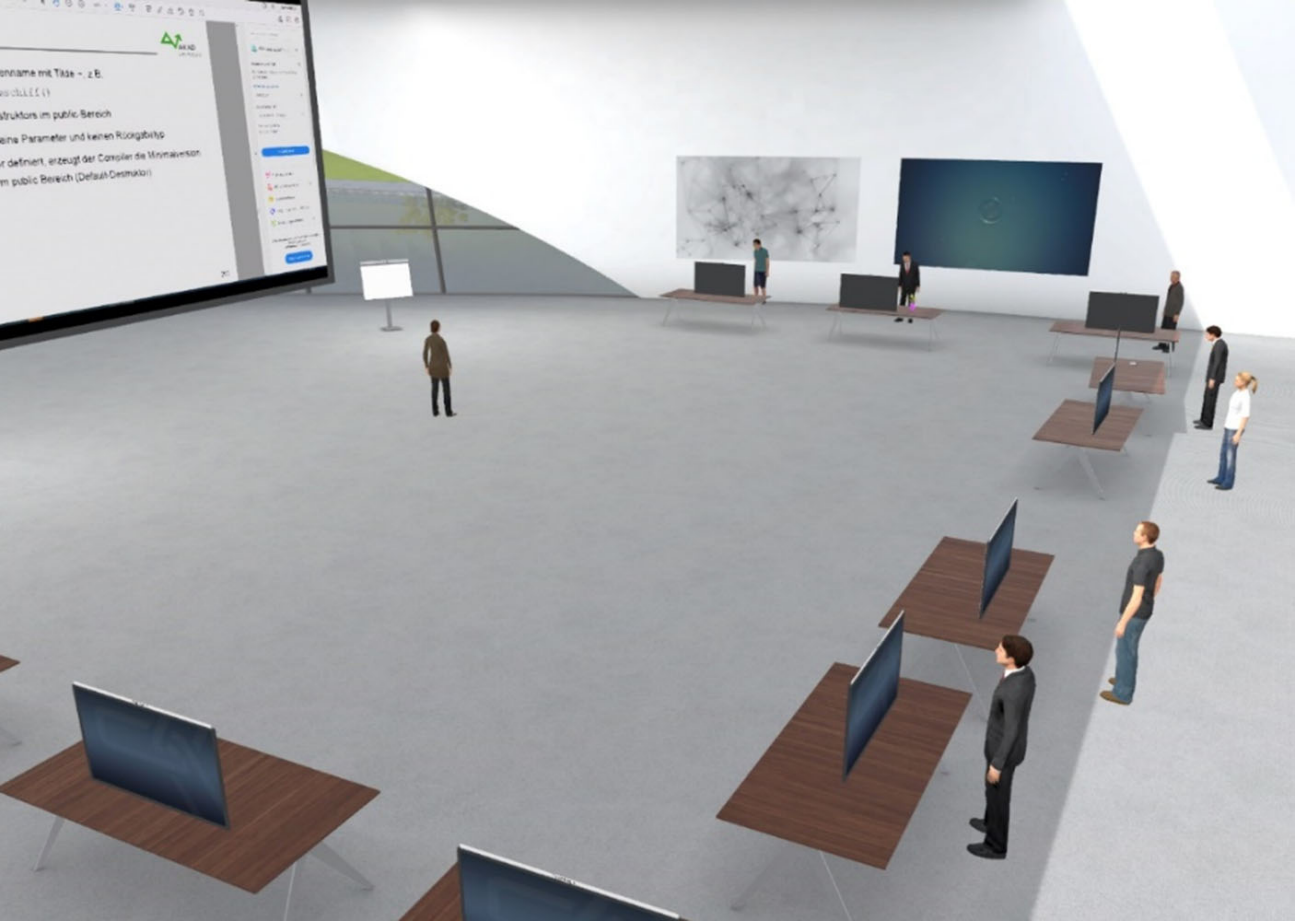
Räumliche Gestaltung des Programmierseminars

Für das *TPS* umfasste die räumliche Gestaltung zusätzliche virtuelle Tische und Monitore in der Aula. Das so entstandene virtuelle Klassenzimmer ermöglichte es jedem der max. 10 Teilnehmer seinen lokalen Bildschirm auf seinem virtuellen Monitor darzustellen (s. Abb. 1). Um Hardware- und Bandbreitenanforderungen zu minimieren, wurden die Bildschirme U‑förmig im Raum platziert, so dass jeder Teilnehmer maximal vier geteilte Bildschirminhalte im Sichtfeld haben kann. Der Dozierende hätte damit die Möglichkeit, analog zur Ausführung in Präsenz, bei Bedarf auf die Programmierumgebung der Teilnehmenden zu sehen und ggfs. zu unterstützen. Des Weiteren würde auch ein Abschauen zwischen den Teilnehmern möglich werden, ein durchaus erwünschter Effekt (Lückemeyer 2015).

**Abb. 2:**
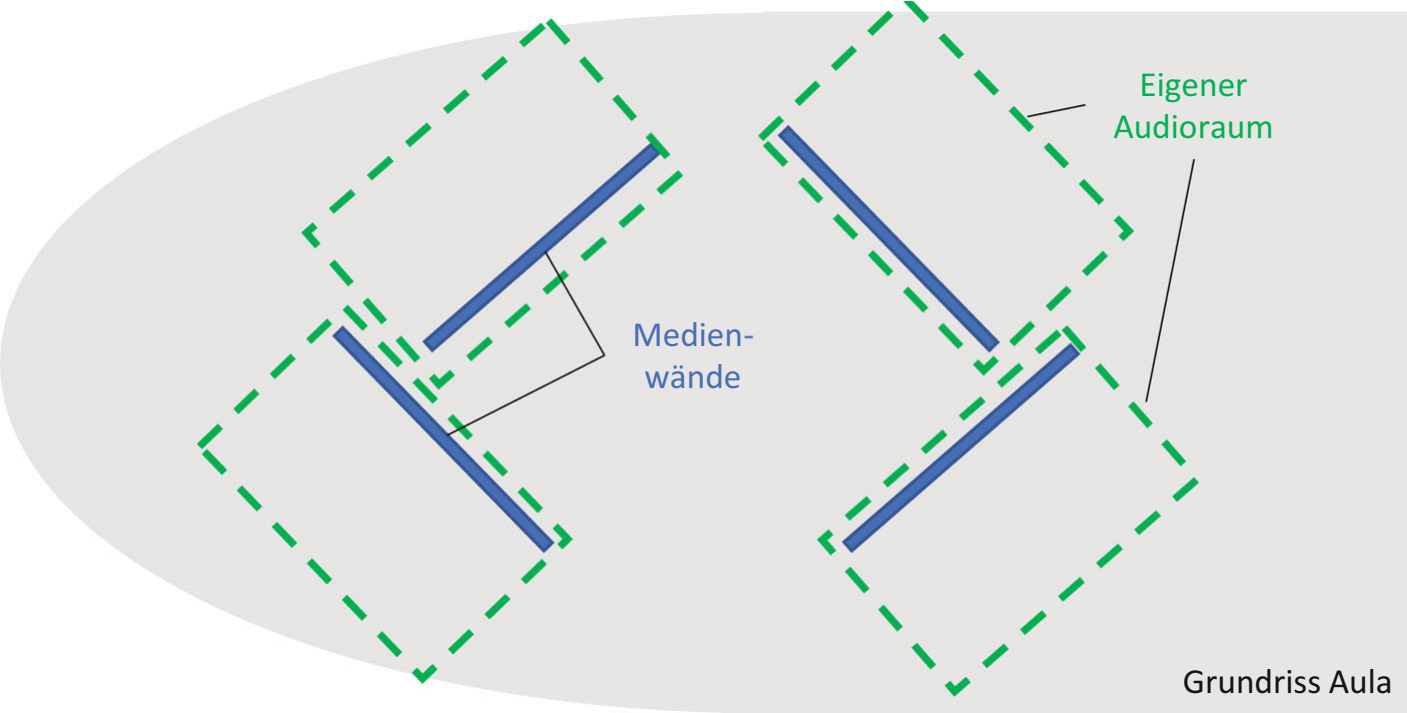
Schematische Darstellung der Raumaufteilung für die Gruppenarbeit

Für das WPS wurde der größte der drei verfügbaren Besprechungsräume mit vier Stehtischen (einen für jede Gruppe) versehen und ein Schild mit der Gruppennummer darauf platziert. Dieser Raum wurde für die Lehranteile in der Großgruppe vorgesehen. Für die Arbeit in Kleingruppen wurde die Aula um Medienwände (Bildseite nach außen) ergänzt und mit einem getrennten Audio- und Videobereich versehen (s. Abb. 2), um dem Wettbewerbscharakter des Planspiels gerecht zu werden. Die Avatare können sich weiterhin sehen, da der Audiobereich nur durch virtuelle Laserstrahlen optisch sichtbar gemacht wird. Der Vorteil dieser Lösung gegenüber der Entsendung in die Besprechungsräume ist, dass die Wege kürzer sind. Das erleichtert dem Dozierenden die schnelle Unterstützung und inkludiert Teilnehmer, die mit der Avatar-Navigation schwer zurechtkommen. Die TopSim-Anwendung sollten die Teilnehmer auf dem *Cloud Desktop* der Medienwand ausführen. Diese virtuellen Maschinen ermöglichen ein gemeinsames Arbeiten an einem Rechner, die Planspielanwendung sollte auf dem dortigen Browser ausgeführt werden. Andere Mitglieder können die Steuerung übernehmen, um selbst aktiv zu werden. Teil der Evaluierung war es herauszufinden, ob es gelingt, dass sich mehr Studierende nicht nur verbal beteiligen, sondern auch die Simulation bedienen.

Den eigentlichen Lehrveranstaltungen wurde 2–3 Tage vorher ein synchroner Technik-Test vorgeschaltet und vorab per E‑Mail kommuniziert. Die Teilnahme war freiwillig, um die zumeist berufsbegleitend Studierenden durch den Zusatztermin nicht zu verärgern. Für Zoom gibt es im Learning Management System Manuale und Erklärvideos, der Support wird standardmäßig über die Studienbetreuung abgedeckt. Die 3D-Lehrveranstaltungen wurden jeweils von einer wissenschaftlichen Mitarbeiterin zur Beobachtung, sowie einer Supportkraft der TriCAT GmbH für die technische Unterstützung begleitet.

## Evaluierung und Ergebnisdiskussion

Zunächst wird in der Ergebnisdarstellung auf die Teilnehmerverteilung und den Funktionenvergleich von 2D zu 3D eingegangen. Anschließend werden die beobachteten und per Fragebogen erhobenen Unterschiede dargestellt und diskutiert (Tab. 1).

**Tab. 1:** Teilnehmer und Rücklaufquote

	TPS		WPS	
	2D	3D	2D	3D
*Veranstaltungsteilnehmer*	7	7	8	8
*Befragungsteilnehmer*	5	5	7	6
*Teilnahmequote Befragung*	71 %	71 %	88 %	75 %

Beide Seminartypen (TPS und WPS) wurden jeweils in TriCAT spaces und in Zoom umgesetzt, bei ansonsten gleichen Parametern. In Tab. 2 werden nur die genutzten Funktionen gegenübergestellt und im Anschluss punktuell erläutert.

**Tab. 2:** Gegenüberstellung der genutzten Funktionen in 2D und 3D

Eingesetzte Funktion	2D	3D
*Installation*	Optional	Obligatorisch
*Login*	Nicht notwendig	Obligatorisch
*Repräsentation der Teilnehmer*	Videobild (optional)	Avatar
*Mimik*	Per Videobild	Automatische Lippensynchronisation
*Gestik*	Teilweise per Videobild	Automatisch beim Sprechen
*Raumwechsel*	Per Knopfdruck	Per Avatar-Navigation
*Raumgestaltung*	Nicht möglich	Innen- & Außenbereich; Anpassungen per Editor
*Interaktion*	Emojis	Avatar-Interaktion (z. B. Handheben)
*Umfragefunktion*	Vorhanden	Vorhanden
*Cloud Desktop*	Nicht vorhanden	Einer je Gruppe
*Bildschirmteilen*	Einer zeitgleich	Alle zeitgleich

Die Zoom-Teilnahme und Authentifizierung erfolgt über einen personalisierten Link im Learning Management System. Eine Teilnahme ist auch per Web-App möglich, d. h. die Installation der Zoom-Software ist optional. Anders bei den 3D-Kursen, die zwingend eine Installation erfordern, sowie eine Authentifizierung über einen Zugangscode, den die Teilnehmer vorab per E‑Mail erhalten. Während es bei einer Videokonferenz per se keine Raumgestaltung gibt, ist deren Gestaltung bei Veranstaltungen in 3D obligatorisch. Dabei kann zwischen den Räumen im Bürogebäude auch der Außenbereich genutzt werden, was im WPS auch für einen Vorlesungspart spontan erfolgte. Alle Teilnehmer waren bereits angemeldet und sind von der üblichen Ausführung in Zoom ausgegangen. Um Teilnehmer nicht zu dem Mehraufwand durch Installation und Einarbeitung in TriCAT spaces zu zwingen, wurden zeitnahe Alternativtermine für Zoom angeboten. Allerdings hat keiner der angemeldeten Teilnehmer von diesem Angebot Gebrauch gemacht, so dass die Teilnehmer repräsentativ sind.

Die vorab festgelegten Beobachtungskriterien für die 3D-Seminare waren die tatsächliche Teilnehmerzahl, die Supportintensität beim Login, Anzahl technisch bedingter Verspätungen, Teilnahmequote am Technik-Test, die Beteiligung unterschiedlicher Benutzer an den Cloud Desktops (nur WPS), sowie Teilen des Programmierbildschirms (nur TPS). Diese Kriterien sind für die Zoom-Seminare nicht relevant, da Zoom über eine Schnittstelle zum Learning Management System verfügt und damit technisch wie organisatorisch integrativer Bestandteil des heutigen Studienmodells ist (Tab. 3).

**Tab. 3:** Beobachtungsergebnisse 3D-Lehrveranstaltungen nach Zielgruppe

Beobachtungskriterium	TPS	WPS
*Seminarteilnehmeranzahl*	7	8
*Teilnehmer mit Support >* *2* *min*	0	3
*Verspätungen >* *10* *min*	Keine	Keine
*Teilnahmequote Technik-Test*	57 %	70 %
*Cloud Desktop Nutzerwechsel*	n. r.	Keiner
*Teilen des Bildschirms*	Nur situativ und vereinzelt	n. r.

Die Beobachtung zeigte einen höheren Supportaufwand bei der nicht-technischen Zielgruppe bei gleichzeitig höherer Teilnahmequote beim Technik-Test. Die Hauptprobleme lagen bei der Eingabe der Logindaten, die der automatisiert generierten Einladungs-E-Mail zu entnehmen sind, sowie an einem fehlenden Headset und damit verbundener Echoentwicklung. Alle drei Probleme traten bereits beim Technik-Test auf und konnten bis zum WPS gelöst werden. D. h. beide Veranstaltungen (WPS und TPS) konnten wie geplant starten, es gab keine technisch bedingte Verzögerung. Bei den *Cloud Desktops* war kein Nutzerwechsel zu beobachten. Da sich alle Studierenden aktiv an der Gruppendiskussion beteiligten, könnten die Gründe dafür auch in der Usability liegen, oder an der Tatsache, dass dem aktuellen Bearbeiter die Rechte entzogen werden müssten, um selbst die Bearbeiter-Rolle einzunehmen. Die technische Möglichkeit einer gleichzeitigen, gleichberechtigten Bearbeitung könnte die Kollaboration verbessern. Eine detailliertere, ggfs. spielerische Einweisungen in die Funktionen und Bedienung der *Cloud Desktops* könnte technische Hürden reduzieren. Im TPS erfolgte bei Freiwilligkeit des Bildschirmteilens nur in Einzelfällen eine situative Nutzung (s. Abb. 1). D. h. die gewünschten Effekte (Abschauen und Unterstützen) wurden nicht erzielt. Abhilfe schaffen könnte eine spielerische Gruppenarbeit zu Veranstaltungsbeginn, die zum Ziel hat, die Funktion intensiver zu erproben.

Die Online-Befragung umfasste 10 validierte Items zum Flow-Erleben (Rheinberg et al. 2019), die Einschätzung der jeweiligen Online-Variante als Alternative zu Präsenzveranstaltungen, die technische Affinität (6 Items) (Cobb et al. 2009), sowie demografische Daten. Die technische Affinität fördert das Selbstvertrauen im Umgang mit Technik und insbesondere der Vorerfahrung mit Videospielen wird eine positive Wirkung auf die Zufriedenheit im Umgang mit virtuellen Welten im Lernkontext zugeschrieben (Shonfeld und Greenstein 2021). 3D-Teilnehmenden wurde zusätzlich die binäre Frage gestellt, welche Veranstaltungssoftware sie bevorzugen. Diese binäre Frage wurde von der Geschäftsleitung der privaten Hochschule als Entscheidungsgrundlage zur Weiterverfolgung des Projektes gefordert. Die Befragung fand zwischen Dezember 2021 und Februar 2022 statt und wurde mit *Umfrageonline* durchgeführt. Die statistische Auswertung erfolgte mit der freien Software *Jamovi*.

Das Durchschnittsalter der in Tab. 1 aufgeschlüsselten Teilnehmer lag bei rund 31 Jahren (Min. 22; Max. 53) und bildet die Altersstruktur der Institution treffend ab. Bei den 3D-Seminaren waren sieben Teilnehmende männlich und vier weiblich, beim 2D-Seminar waren es je sechs Teilnehmende pro Geschlecht. Das Flow-Empfinden wurde per 8‑stufiger Likert Skala (1 = trifft nicht zu; 8 = trifft zu) gemessen. Im Durchschnitt zeigte sich nur ein leicht höheres Flow-Empfinden bei der 3D-Veranstaltung (3D_MW: 5,45; 3D_SD 1,77; 2D_MW: 5,33; 2D_SD 1,69). Die Standardabweichung deutet auf individuelle Unterschiede hin und unterstützt bisherige Forschungsergebnisse, die Abhängigkeiten z. B. vom Alter identifizierten (Faiola et al. 2013). Die Reliabilitätsanalyse lieferte einen Wert für Cronbachs Alpha von als sehr gut zu bewertenden 0,862, so dass davon ausgegangen werden kann, dass die interne Konsistenz und damit die Messgenauigkeit für Flow gegeben sind.

Neun (davon fünf aus dem WPS und vier aus TPS) von elf befragten 3D-Seminarteilnehmenden (82 %) bevorzugen TriCAT Spaces gegenüber Zoom. Insgesamt werden beide Online-Varianten als sehr gute Alternative zu Präsenzveranstaltungen gewertet (3D_MW 6,36; 3D_SD 0,92; 2D_MW 6,58; 2D_SD 0,9; 1 = trifft nicht zu; 7 = trifft zu). Die hohe Akzeptanz von Onlineveranstaltungen war unter Studierenden, die sich für ein digitales Fernstudium entschieden haben, erwartbar. Die durchschnittliche technische Affinität wurde erfasst, um die Präferenz für die virtuelle Welt und die Unterstützungsintensität für die unterschiedlichen Zielgruppen (technisch und nicht-technisch) zu ergründen, da angenommen wurde, dass sich diese Zielgruppen hinsichtlich der technischen Affinität unterscheiden. Die gemessenen Unterschiede fielen allerdings gering aus. So zeigte sich über alle sechs Items für die Programmierer (MW 5,80; SD 1,00) nur ein geringfügig höherer Durchschnittswert als bei den Ökonomen (MW 5,54; SD 0,92), ist aber bei beiden Zielgruppen hoch. Auffällig war das Item „Ich spiele ziemlich oft Computerspiele“. Durch das Entfernen dieses Items ließ sich Cronbachs Alpha von 0,657 auf 0,728 erhöhen, so dass die Programmierer (MW 6,24; SD 0,73) nur noch eine minimal höhere technische Affinität zeigten als die Ökonomen (MW 6,17; SD 0,74). Dennoch wurden bei den Ökonomen höhere Supportaufwände beobachtet als bei den Programmierern. Ein möglicher Grund dafür liegt ggfs. in der Selbstbeurteilung der eigenen technischen Fähigkeiten. Da das Spielen von Videospielen als Einflussfaktor auf die Zufriedenheit mit der virtuellen Welt zu Bildungszwecken gilt, wurde es im Folgenden nicht entfernt, sondern getrennt von der technischen Affinität betrachtet. Im Vergleich zur technischen Affinität fiel die Videospielhäufigkeit allgemein niedrig aus, bei hohen individuellen Unterschieden und einer deutlichen Differenz zwischen den Zielgruppen (siehe Tab. 4).

**Tab. 4:** Technische Affinität und Videospielhäufigkeit nach Zielgruppe (1 = trifft nicht zu; 7 = trifft zu)

	Seminar	*N*	MW	SD
Ich bin sicher im Umgang mit Computern für grundlegende Aufgaben	TPS	10	6,80	0,42
	WPS	13	6,77	0,44
Ich bin zuversichtlich, dass ich mich in neue Software einarbeiten kann	TPS	10	6,90	0,32
	WPS	13	6,46	0,78
Das Internet ist eine hilfreiche und unterhaltsame Ressource	TPS	10	6,30	0,95
	WPS	13	6,08	1,44
Ich habe Angst vor der Benutzung von Computern. (invertiert)	TPS	10	6,90	0,32
	WPS	13	6,77	0,60
Ich fühle mich durch das Internet eingeschüchtert. (invertiert)	TPS	10	6,30	1,64
	WPS	13	6,77	0,44
Ich spiele ziemlich oft Computerspiele	TPS	10	3,60	2,37
	WPS	13	2,38	1,81

Trotz der geringen Verbreitung von Videospielern unter den Befragten, bevorzugten der Großteil der Teilnehmenden in 3D diese Online-Methode. Es kann vermutet werden, dass die berufsbegleitend Studierenden keine Zeit für Videospiele haben, aber aus ihrem Berufsalltag videokonferenzmüde geworden sind und Abwechslung und Innovation schätzen. Die hohe technische Affinität auch bei wirtschaftlichen Studiengängen wird auf das digitale Studienmodell zurückgeführt, kann aber nicht automatisch auf weitere Zielgruppen beispielsweise aus dem sprachlichen oder sozialen Bereich übertragen werden.

## Zusammenfassung und Ausblick

Die im Zuge der Corona-Pandemie festgestellten Schwächen bei Seminaren über Videokonferenz, wie beispielsweise mangelnde Aktivität und fehlende Unterstützungsmöglichkeiten, sollten über eine Online-Alternative abgemildert werden. Aufgrund der räumlichen und sozialen Präsenz von virtuellen Welten im Allgemeinen und der positiven Erfahrungen in der Hochschullehre mit TriCAT spaces im Speziellen, entstand die Idee diese 3D-Variante gegen die bisherige Online-Seminar-Lösung Zoom zu evaluieren. Zwei Seminare, bei denen die bisherigen Nachteile besonders stark ausgeprägt waren und unterschiedliche Zielgruppen (Wirtschaft und Technik) adressieren, wurden dafür ausgewählt und umgesetzt. Diese beiden Seminare, Wirtschaftsplanspiel und Programmierseminar, wurden in beiden Online-Methoden inhaltlich identisch, aber mit Anpassung an die technischen Möglichkeiten konzipiert und durchgeführt. Die anschließende Evaluierung umfasste neben demographischen Daten im Wesentlichen das Flow-Erleben, die technische Affinität, die Online-Methoden-Präferenz, sowie die Wertung als Präsenzalternative.

Bei der Durchführung des 3D-Planspiels konnte beobachtet werden, dass die *Cloud Desktops* nicht die schriftliche Bearbeitung aller Gruppenmitglieder erreichen konnten, wenngleich alle intensiv mitgearbeitet und diskutiert haben. Im 3D-Programmierseminar wurde der Bildschirm nur situativ von den Teilnehmenden freiwillig freigegeben. Eine gezielte Integration in den Anfang der Agenda zum Erlernen der Funktion in Form einer Gruppenübung oder eines Spiels könnte die technische Hürde zur Nutzung herabsetzen.

Das Ergebnis der Evaluierung (*N* = 23) zeigte ein nur leicht höheres Flow-Erleben in der virtuellen Welt mit deutlichen individuellen Unterschieden und hohem Niveau für beide Online-Varianten. Trotzdem bevorzugte die große Mehrheit die virtuelle Welt, was ggfs. auf die Videokonferenzmüdigkeit in Zeiten von Home-Office zurückzuführen ist. Die Präferenz für Online im Vergleich zu Präsenz ist deutlich. Die technische Affinität ist bei beiden Zielgruppen, Wirtschaft und Technik, ausgesprochen hoch, trotzdem benötigten die Wirtschaftsstudierenden mehr technischen Support. Das regelmäßige Spielen von Videospielen ist unter den befragten Fernstudierenden nicht verbreitet, scheint aber keine Grundvoraussetzung für die Bevorzugung der 3D-Variante zu sein. Genauere Ursache-Wirkung-Aussagen ließen sich machen, wenn bei zukünftigen Befragungen die Videospielerfahrung, statt der aktuellen Videospielhäufigkeit, abgefragt würde. Die Ergebnisse lassen sich auf andere Fernhochschulen, aber auch Präsenzhochschulen übertragen. Ein Transfer auf weitere Zielgruppen ist nicht ohne Weiteres möglich.

Die Stichprobe ist klein und die Ergebnisse daher nicht repräsentativ. Dennoch lassen sich Implikationen für Forschung und Praxis ableiten. Ein Forschungsfeld eröffnen die räumliche und methodische Gestaltung von 3D-Lehrveranstaltungen. Das Flow-Konstrukt zum Vergleich des Potenzials der unterschiedlichen Methoden zeigte keine eindeutigen Unterschiede und ist damit ggfs. für künftige Forschungen zu hinterfragen. Bei einer regelmäßigen praktischen Nutzung von virtuellen Welten für die digitale Lehre stellen die höheren Supportaufwendungen eine personelle Herausforderung dar. Innovative Lösungsansätze aus der Forschung, wie beispielsweise die auf künstlicher Intelligenz basierenden Sprachassistenten, könnten die Supportaufwände reduzieren und wiederum Einfluss auf die Praxis nehmen (Khosrawi-Rad et al. 2022). Darüber hinaus können weitere Zielgruppen und Lehrveranstaltungen, wie beispielsweise Labore, einbezogen werden. Neben der reinen Inhaltsvermittlung könnte der Austausch zwischen Studierenden zur sozialen Integration ein interessanter Anwendungsfall im Fernstudium sein. Für die Praxis lässt sich ableiten, dass virtuelle Welten einen Entwicklungsstand erreicht haben, der auch für nicht-technische Zielgruppen in der Hochschulbildung, aber ggfs. auch darüber hinaus in der beruflichen und innerbetrieblichen Weiterbildung, einsetzbar ist.
